# Emerging directions in tauopathy research

**DOI:** 10.1002/alz.71431

**Published:** 2026-06-21

**Authors:** Luc Buée, Kristin R. Wildsmith, Suvarna Alladi, Taylor Bertucci, Delphine Boche, Kathryn R. Bowles, Adam Boxer, Jennifer Brummet, Sarah L. DeVos, Kristophe Diaz, Anna Jane Dreyer, Karen Duff, Claudia Duran‐Aniotz, Claire S. Durrant, Kurt Farrell, Igor C. Fontana, Nicolai Franzmeier, Bess Frost, Kanta Horie, Petr Kaňovský, Aimee W. Kao, Simin Mahinrad, Maura Malpetti, Huw R. Morris, Carla Palleis, Leonard Petrucelli, Jessica Rexach, Larisa Reyderman, Amy Rommel, James B. Rowe, Sandra M. Sanabria Bohórquez, Chihiro Sato, Michael Schöll, Anja Schneider, Rohan de Silva, Heather M. Snyder, Lawren VandeVrede, Andrew W. Varga, Jacob W. Vogel, Felix L. Yeh, Andrew S. Yoo, Maria C. Carrillo

**Affiliations:** ^1^ Lille Neuroscience and Cognition Univ. Lille, Inserm CHU‐Lille, Lille France; ^2^ Eisai Inc. Nutley New Jersey USA; ^3^ National Institute of Mental Health and Neurosciences Bengaluru India; ^4^ Neural Stem Cell Institute Albany New York USA; ^5^ Clinical Neurosciences, Clinical and Experimental Sciences, Faculty of Medicine University of Southampton Southampton UK; ^6^ UK Dementia Research Institute University of Edinburgh Edinburgh Scotland UK; ^7^ Institute for Neuroscience and Cardiovascular Research University of Edinburgh Edinburgh Scotland UK; ^8^ Edward and Pearl Fein Memory and Aging Center, Weill Institute of Neuroscience, Department of Neurology University of California, San Francisco San Francisco California USA; ^9^ CurePSP New York New York USA; ^10^ Curie.Bio Boston Massachusetts USA; ^11^ Department of Clinical Neurosciences University of Cambridge Cambridge UK; ^12^ UK Dementia Research Institute University College London London UK; ^13^ Latin American Brain Health Institute (BrainLat) Universidad Adolfo Ibanez Santiago Chile; ^14^ Global Brain Health Institute (GBHI) Trinity College Dublin (TCD) Dublin Ireland; ^15^ Department of Pathology Icahn School of Medicine at Mount Sinai New York New York USA; ^16^ Department of Artificial Intelligence & Human Health Icahn School of Medicine at Mount Sinai New York New York USA; ^17^ Nash Family Department of Neuroscience Icahn School of Medicine at Mount Sinai New York New York USA; ^18^ Ronald M. Loeb Center for Alzheimer's Disease Icahn School of Medicine at Mount Sinai New York New York USA; ^19^ Friedman Brain Institute Icahn School of Medicine at Mount Sinai New York New York USA; ^20^ Neuropathology Brain Bank & Research CoRE Icahn School of Medicine at Mount Sinai New York New York USA; ^21^ Alzheimer's Association Chicago Illinois USA; ^22^ Institute for Stroke and Dementia Research (ISD) LMU University Hospital, Ludwig‐Maximilians‐Universität (LMU) Munich Germany; ^23^ Munich Cluster for Systems Neurology (SyNergy) Munich Germany; ^24^ Department of Psychiatry and Neurochemistry, Institute of Neuroscience and Physiology, Sahlgrenska Academy University of Gothenburg Mölndal Sweden; ^25^ Center for Alzheimer's Disease Research Brown University Providence Rhode Island USA; ^26^ Department of Neurology Washington University School of Medicine St. Louis Missouri USA; ^27^ The Tracy Family SILQ Center Washington University School of Medicine St. Louis Missouri USA; ^28^ Department of Neurology and Clinical Neuroscience Center University Hospital Olomouc Olomouc Czech Republic; ^29^ Department of Neurology, Faculty of Medicine and Dentistry Palacky University Olomouc Czech Republic; ^30^ Department of Clinical Neurosciences University of Cambridge and Cambridge University Hospitals NHS Trust, University of Cambridge Cambridge UK; ^31^ UK Dementia Research Institute at University of Cambridge Cambridge UK; ^32^ Movement Disorders, UCL Queen Square Institute of Neurology London UK; ^33^ National Hospital for Neurology and Neurosurgery, Queen Square London UK; ^34^ Department of Neurology LMU University Hospital, Ludwig‐Maximilians‐Universität (LMU) Munich Germany; ^35^ German Center for Neurodegenerative Diseases (DZNE) Site Munich Munich Germany; ^36^ Department of Neuroscience Mayo Clinic Jacksonville Florida USA; ^37^ Department of Neurology, David Geffen School of Medicine University of California Los Angeles California USA; ^38^ Rainwater Charitable Foundation Fort Worth Texas USA; ^39^ Department of Clinical Neurosciences MRC Cognition and Brain Sciences Unit and Cambridge University Hospitals NHS Trust, University of Cambridge Cambridge UK; ^40^ Imaging Data Insights, Research & Early Development, Genentech, Inc. South San Francisco California USA; ^41^ Wallenberg Centre for Molecular and Translational Medicine University of Gothenburg Gothenburg Sweden; ^42^ Department of Neuropsychiatry Sahlgrenska University Hospital Region Västra Götaland, Mölndal Sweden; ^43^ German Center of Neurodegenerative Diseases, DZNE Bonn Germany; ^44^ Department of Old Age Psychiatry and Cognitive Disorders University Hospital Bonn Bonn Germany; ^45^ Reta Lila Weston Institute and Department of Clinical & Movement Neurosciences UCL Queen Square Institute of Neurology London UK; ^46^ David M. Rapoport Sleep Research Laboratory, Division of Pulmonary, Critical Care, and Sleep Medicine, Friedman Brain Institute Icahn School of Medicine at Mount Sinai New York New York USA; ^47^ Department of Clinical Sciences SciLifeLab, Lund University Lund Sweden; ^48^ Translational Medicine, Genentech, A Member of the Roche Group South San Francisco California USA; ^49^ Department of Developmental Biology Washington University School of Medicine St. Louis Missouri USA

**Keywords:** biomarkers, neurodegeneration, precision medicine, tauopathies, therapeutics

## Abstract

The Tau Global Conference 2025, hosted by the Alzheimer's Association, CurePSP, and the Rainwater Charitable Foundation, convened international experts from academia, industry, government, and philanthropy to explore advances and challenges in tauopathy research. The meeting highlighted progress across tau biology, including emerging models of tau regulation, degradation, and propagation; advances in biomarker development for the diagnosis and staging of tauopathies; and evolving therapeutic strategies targeting diverse aspects of tau pathophysiology. Discussions also emphasized the importance of cross‐sector collaboration, and global initiatives to address disparities in tau research. This report synthesizes key insights from the conference and underscores the critical role of interdisciplinary, biomarker‐driven, and globally inclusive approaches in accelerating the translation of tau research into effective clinical applications.

## Highlights

- The Tau Global Conference 2025 showcased recent advances across tauopathy biology, biomarkers, and therapeutics.
- Emerging experimental models and biomarker platforms are refining disease understanding, diagnosis, and staging of tauopathies.
- Biomarker‐informed and precision trial designs are reshaping tau‐targeted therapeutic development.
- Global and interdisciplinary collaboration is essential to address disease heterogeneity and research inequities in tauopathies.

## 1 BACKGROUND

Tau plays an important role in a wide range of neurodegenerative diseases, collectively known as tauopathies, and understanding its function and dysfunction is central to improving diagnosis, monitoring, and treatment across these disorders. Tauopathies include primary tauopathy disorders in which tau pathology represents the predominant neuropathological hallmark, such as progressive supranuclear palsy (PSP), corticobasal degeneration (CBD), Pick's disease, and frontotemporal dementia with tau pathology (FTLD‐tau). Secondary tauopathy disorders feature tau abnormalities alongside other pathological processes, as seen in Alzheimer's disease (AD), Lewy body dementia (LBD), and Parkinson's disease (PD). Research in the field of tauopathies has been complicated due to several factors such as the molecular complexity of tau protein and its fibrillary forms, the clinical and pathological heterogeneity of tauopathies, the absence of reliable early biomarkers, and the limited translation of preclinical discoveries into clinical interventions.

To address these gaps and accelerate progress, the Tau Global Conference 2025 (held in London and online in April 2025) brought together experts from academia, industry, philanthropy, government, and persons with lived experience. The meeting served as a global state‐of‐the‐science forum, exploring cutting‐edge advances in tau‐related biology, biomarkers, therapeutics, and phenotypes; fostering talent development; and promoting interdisciplinary collaboration to address persistent challenges in the field. The meeting also deeply valued the voices and lived experiences of those living with any disease that causes tauopathy and their care partners. A panel discussion featuring individuals living with PSP, CBD, and genetic FTD highlighted personal diagnostic journeys, the day‐to‐day impact of these conditions, and the challenges faced by families and care partners. Speakers emphasized the critical role of advocacy, raising awareness, and advancing research efforts to improve outcomes for affected individuals, care partners, and future generations.

The Tau Global Conference 2025 represented the merger of three major tau‐focused conferences—Global Tau, EuroTau, and CurePSP Neuro—and was hosted by the Alzheimer's Association, CurePSP, and the Rainwater Charitable Foundation.

This manuscript presents a summary of the scientific content and key insights from the meeting with the goal of providing the field with a structured, accessible record of current advances and outstanding questions in tau research, while reflecting the breadth of perspectives and approaches shared at the conference.

## 2 TAU STRUCTURE, FUNCTION, AND EMERGING EXPERIMENTAL PLATFORMS

Tauopathies arise from the disruption of the tau protein's delicate physiological balance, tipping the scale from its normal functions to pathogenic aggregation. This shift has driven extensive research into the molecular mechanisms that regulate tau's normal activity, including its role in stabilizing microtubules, maintaining axonal transport, and supporting neuronal integrity. Equally critical is understanding the processes by which tau undergoes pathological transformation, leading to misfolding, hyperphosphorylation, and self‐assembly into conformationally distinctive fibrillar aggregates.

Recent advances illuminate the structural domains of tau, its post‐translational modifications, and the cellular pathways that are involved in clearance and degradation. In addition, emerging experimental platforms are enabling increasingly precise modeling of tau homeostasis, propagation, and disease mechanisms. Together, these insights provide a foundation for linking molecular biology to clinical phenotypes, and for developing innovative therapeutic strategies that target the tau pathway at multiple levels.

### 2.1 Lysosomal enzyme contributions to tau homeostasis

Tau exists in a delicate balance between production and clearance, and between its physiological roles and pathological modifications. Lysosomal biology has emerged as a key determinant of tau homeostasis. The lysosome is a degradative organelle responsible for clearing tau and other damaged or misfolded proteins from the cell. Lysosomal dysfunction has been heavily implicated in the pathogenesis of neurodegenerative diseases generally, and in tauopathies specifically.^1^ Lysosomal proteases, collectively referred to as cathepsins, exhibit selective, pH‐dependent degradation of tau. While some autosomal dominant coding mutations in the tau gene (microtubule‐associated protein tau [*MAPT])* lead to tau self‐association,^2^, ^3^ whether they can also alter the ability of lysosomes to degrade tau has not been explored.

To address this gap, researchers have generated a lysosomal protease cleavage map of tau using in silico analyses of cleavage motifs and in vitro protease assays.^4^ It demonstrated that many cathepsins can cleave tau in regions where disease mutations map. Using in silico predictions and in vitro protease assays, it was shown that many *MAPT* coding mutations diminish or completely abrogate tau cleavage at specific sites, and that the half‐life of mutant tau (e.g., K257T, N279K and S305N tau) is prolonged in cells and/or induced pluripotent stem cell (iPSC) ‐derived neurons. Over decades, as aging renders lysosomes less efficient, this could lead to increased steady‐state levels of tau, which in turn promote tau fibril formation and neurodegeneration.^4^

The tau lysosomal protease map also revealed that a region of tau, the proline‐rich domain, is relatively resistant to proteolytic cleavage. Interestingly, the proline‐rich domain of tau contains threonine residues at positions 181 and 217, which, as phospho‐peptides, are United States Food and Drug Administration (FDA)‐approved cerebrospinal fluid (CSF) and plasma biomarkers for AD.^5^ Recent findings indicate that tau phosphorylation at these sites further enhances proteolytic resistance, rendering these peptides even less susceptible to cleavage.^6^ The level of ptau‐181 in the CSF of AD subjects correlates with those of CSF lysosomal proteases that reach the extracellular space through lysosomal exocytosis.^6^ Therefore, we speculate that the proteolytic resistance of p‐tau 181 and 217 peptides may facilitate their release into the CSF and plasma, thereby contributing to their utility as fluid AD biomarkers. These recent findings provide a potential biological rationale for why tau phospho‐peptides persist and are available as AD biomarkers.

Taken together, the lysosome is a critically important sub‐cellular organelle responsible for tau degradation and clearance. Its age‐associated decline, along with the remarkable specificity of tau cleavage by cathepsins, likely contribute to the mechanism whereby autosomal dominant disease mutations in *MAPT* may contribute to mechanisms underlying tauopathies. The inability of lysosomes to cleave p‐tau in the proline‐rich domain may also result in release of p‐tau 181 and p‐tau 217 as biomarkers. This nominates the lysosome as a promising target for future research and therapeutic development.

### 2.2 Tau pathology spreading in AD: Neuroimaging and cellular insights

In AD, production of misfolded tau exceeds its clearance, leading to local accumulation. Over time, tau burden becomes more widespread across the brain. This expanding distribution can be partly driven by the propagation of misfolded tau species across brain networks, further amplifying disease burden. Studies in cellular and animal models of tauopathy demonstrate that tau can propagate trans‐synaptically in an activity‐dependent manner across connected neurons contributing to the early spread of pathological tau species before substantial insoluble deposition occurs.^7^ Translating this concept to in vivo human studies, researchers combined tau–positron emission tomography (PET) imaging with measures of brain connectivity to demonstrate that tau pathology in AD spreads preferentially along brain networks.^8^, ^9^, ^10^, ^11^, ^12^ Importantly, this analytic framework applied to mouse models of tauopathy also successfully predicts spreading patterns, providing important cross‐species analogues of connectome‐based models.^13^

This connectivity‐based model of spread could potentially be used to predict individual‐specific trajectories of tau accumulation in people with or at risk of disease.^14^, ^15^ Notably, despite spatial heterogeneity in tau epicenters, their underlying connectivity profiles appear to govern subsequent tau propagation. Tau spread is further accelerated when tau epicenters reside in highly connected hub regions, underscoring the role of brain network topology in modulating disease progression.^10^, ^16^, ^17^ Extending these findings to primary four‐repeat (4R) tauopathies such as PSP, tau deposition patterns were found to similarly follow the brain's functional connectivity structure.^18^ Taken together, these data suggest that connectivity‐associated tau propagation may represent a common mechanism across both primary and secondary tauopathies.

Amyloid‐β (Aβ) is reported to accelerate intercellular spread of tau in AD, and this finding has been implemented into connectome‐based models of tau spread.^19^ Aβ appears to initially induce neuronal hyperactivity, which facilitates tau release and trans‐synaptic spread.^20^ In individuals with AD, neuronal hyperconnectivity^21^ and elevated soluble p‐tau^22^ in biofluids were identified as key risk‐factors linking Aβ pathology to downstream tau tangle formation. This supports a model in which Aβ‐induced network dysfunction initially accelerates the dissemination of tau pathology via activity‐dependent mechanisms, even where tau pathology is subsequently synaptotoxic.^23^ Future studies should explore whether common mechanisms underlie tau propagation across tauopathies to inform broader therapeutic strategies and provide a clearer understanding of the different disease pathophysiology.^24^

Accumulating evidence supports an active role of glial and immune cells in facilitating tau seeding and spread.^25^, ^26^, ^27^ Neuropathological and genetic studies point to Aβ deposition as an initiating event in many models of AD,^28^, ^29^ with tau pathology emerging downstream and acting as a primary driver of neurodegeneration and dementia severity. Genome‐wide association studies (GWAS) implicate microglia as key contributors to disease progression, with emerging evidence suggesting potential roles earlier in disease, including in shaping vulnerability to pathology. These findings support a model in which Aβ deposition (and tau) triggers microglial activation and neuritic dystrophy, rendering neurons vulnerable to tau spread. Pre‐existing p‐tau from the limbic system can then disseminate from dystrophic neurites to neuronal soma, ultimately forming neurofibrillary tangles and propagating through the neocortex in a prion‐like manner, leading to neurodegeneration and cognitive impairment.^30^

Support for this framework comes from human *post mortem* studies showing that, in the absence of clinically diagnosed dementia, microglia often cluster around Aβ plaques with p‐tau–negative dystrophic neurites. In contrast, in brains of people who had dementia, neuritic plaques are characterized by the co‐localization of Aβ, microglia, and p‐tau–positive dystrophic neurites.^31^ This observation is corroborated by another study with several microglial markers strongly associated with tau‐containing neuritic plaques and tangles in AD.^32^ Importantly, in brains of individuals with AD immunized against Aβ, clearance of plaques was associated with restoration of microglial homeostasis and reduced tau pathology.^33^, ^34^, ^35^ Beyond AD, evidence from large *post mortem* studies of frontotemporal lobar degeneration (FTLD) showed associations between p‐tau expression and phagocytic or reactive microglial markers, despite the absence of overt microglial activation.^36^ These studies also implicate altered astrocytic function, particularly in glutamate cycling, as well as recruitment of CD8+ T cells in tau‐related pathology.^36^

Emerging evidence suggests tau accumulation is likely determined by a combination of connectivity and regional vulnerability.^37^, ^38^, ^39^ A comprehensive model of tau spread will therefore need to integrate these different elements, and will likely need to incorporate insights from clinical, preclinical, and *post mortem* work. Computational models have emerged that simulate the spread of neurodegenerative pathology through the brain,^40^ which can be informed by data sourced from living humans to measure brain connectivity and neuropathology.^41^, ^42^ These models can simulate mechanistic features of protein metabolism,^12^ while modeling spread across the whole brain, and incorporating a priori regional vulnerability information to help determine the level of protein accumulation in a given brain area.^12^, ^39^ A recent study applied this modeling framework to tau in AD and suggested that different elements may contribute to the distribution of tau in the human brain.^43^ The overall pattern of tau distribution (i.e., where tau accumulates) appeared to be governed mostly by brain connectivity and the distribution of different neuroreceptor systems. In contrast, regional tau load (i.e., how much tau accumulates in each region) instead depended more on local factors, such as MAPT expression, local Aβ levels, and blood flow volume. While person‐to‐person variation in patterns of tau accumulation are well described,^44^, ^45^, ^46^ the study suggests that this variation may be driven by differences in tau load, rather than differences in where tau spreads.

Taken together, these findings provide new insights into the mechanisms of tau propagation and highlight emerging avenues for advancing our understanding of tauopathies.

### 2.3 Emerging approaches and technologies

#### 2.3.1 Human cell‐based models of AD: Vascular and neuronal reprogramming approaches

Across neurodegenerative diseases, and notably in AD, recent work has highlighted possible interactions between vascular dysfunction and tau pathology.^47^, ^48^ In addition, CSF‐blood borders could also be affected. For instance, tanycytes, ependymal cells at the border of the third ventricle, move tau from CSF to blood and these cells degenerate in AD.^49^ However, the extent, mechanisms, and generalizability of these interactions across tauopathies remain incompletely understood and represent an active area of investigation. Although available data are still limited, several methodological advances are improving our ability to characterize vascular involvement in neurodegeneration. A long‑standing challenge has been the relatively poor capture of vascular cell types in single‑nucleus RNA‑sequencing datasets, which has historically constrained efforts to comprehensively assess vascular alterations in human disease tissue. Large‐scale integrative transcriptomic analyses are now helping to expand donor representation and enhance cellular resolution, while human iPSC‐derived vascular models^50^, ^51^ provide complementary experimental platforms for investigating associated vascular phenotypes.

As an example, elevated fibronectin (FN1) surrounding cerebral blood vessels has been reported in apolipoprotein E4 (APOE4) carriers with AD.^52^ APOE4/4 iPSC‐derived vascular models appear to recapitulate this feature, exhibiting increased FN1 gene expression and protein deposits.^53^, ^54^ Although the mechanistic implications of FN1 dysregulation remain under investigation, the identification of an FN1 loss‐of‐function variant associated with reduced APOE4‐related AD risk.^52^ Together, these advances are beginning to lay the groundwork for a more comprehensive understanding of vascular contributions to neurodegeneration.

Direct neuronal reprogramming^55^ offers a complementary approach for investigating age‐related neuronal vulnerability underlying late‐onset AD (LOAD). This technique converts adult somatic cells, such as fibroblasts, directly into neurons thereby bypassing the rejuvenation associated with pluripotency and retaining aging signatures.^56^, ^57^ A key advance in this approach has been the use of brain‐enriched microRNAs (miRNAs), miR‐9/9* and miR‐124 (miR‐9/9*‐124), which remodels the chromatin landscape, silences fibroblast identity and sequentially activates neuronal gene expression programs.^58^, ^59^, ^60^ Importantly, directly reprogrammed neurons express all six human tau isoforms in a physiological 1‐to‐1 ratio of 3R‐ to 4R‐tau, thus mirroring the adult human brain.^61^ These features make these models particularly useful for studying tauopathies characterized by distinct isoform composition, including 3R/4R tau profiles in AD and 4R‐predominant aggregates in PSP and CBD.^62^ Recent advances have also described iPSC‐derived human neuronal models of elevated 4R tauopathies that develop features of tauopathy, including hyperphosphorylated tau and endogenous aggregates.^63^ When fibroblasts from individuals with LOAD are reprogrammed in a three‐dimensional environment, the resulting neurons develop hallmark AD pathologies, including extracellular Aβ deposition, insoluble tau aggregates, and progressive neuronal loss.^64^

Collectively, iPSC‐derived vascular systems and directly reprogrammed neurons provide complementary platforms to study how vascular dysfunction, genetic risk factors, and cellular aging intersect to drive neurodegeneration. These human cell‐based models bridge the gap between molecular genetics and in vivo pathology, offering new opportunities to identify mechanistic targets and therapeutic strategies for AD and related tauopathies.

#### 2.3.2 MAPT knock‐in mouse models for tauopathies

Mouse models of tauopathy have been used for hypothesis generation and testing, target identification, and therapeutic testing. To better capture human disease features, new knock‐in models have been generated in which the humanized tau (*MAPT*) gene carries mutations that cause frontotemporal dementia (FTD). These lines have also been crossed with amyloid precursor protein (APP) ‐targeted mouse models to produce animals that develop both plaques and tangles. Characterization across multiple levels—including neuropathology, post‐translational modifications (PTMs), tau conformer (seed) formation, and behavioral/cognitive decline—has revealed important mechanistic insights into tau biology.

Mice harboring splice shifting mutations (10+3 and S305N) accumulate hyperphosphorylated tau by 15 months of age, with phospho‐epitopes concentrated in the proline rich domain. These mice lose synapses and develop cognitive and behavioral decline, yet fail to develop detectable aggregates or seed‐competent oligomers of tau even up to 30 months of age.^65^ Comparative studies involving additional *MAPT* knock‐in lines, with and without *APP* mutations, as well as cell‐based models carrying mutations such as P301S, S305N, S320F, and others,^66^, ^67^ highlight divergent pathogenic pathways. Such comparisons demonstrate that distinct forms of pathogenic tau could drive tauopathy and functional decline through different mechanisms, providing valuable systems to dissect the earliest stages of disease and test targeted interventions.

#### 2.3.3 Live human brain slice cultures to investigate tauopathies

Living human brain slice cultures (HBSCs) offer a unique opportunity to study physiological and pathological tau mechanisms in the live adult human brain.^68^, ^69^, ^70^ Using waste tissue generated as a byproduct of tumor debulking surgeries, researchers have shown that slices of living human cortex retain neurons, astrocytes, microglia, and detectable neuronal activity for at least 7 days in vitro.^71^ Sampling of proteins released from HBSCs into culture medium revealed that Aβ1‐40 declined with increasing age, while total tau protein remained stable. Notably, tau concentrations are higher in temporal lobe–derived cultures compared with frontal lobe samples, despite consistent Aβ levels, suggesting regional differences in vulnerability to tau pathology.

Aβ plaques and tau tangles were detected in HBSCs from some older individuals, offering the opportunity to observe how these features impact experimental readouts in live human brain tissue.^71^ In experimental studies, treatment of HBSCs with soluble tau extracts from PSP brain tissue induced significant uptake of oligomeric tau into postsynaptic compartments, along with pronounced astrogliosis and synapse engulfment.^72^ This highlights a mechanism by which tau pathology may spread through the brain. Thus, HBSCs represent a promising but underutilized model for mechanistic research in tauopathies and could fill a crucial gap between preclinical models and human disease.

## 3 GENETIC AND ENVIRONMENTAL DRIVERS OF TAUOPATHIES

Genetic and environmental factors shape the risk, progression, and clinical variability of tauopathies. Tau pathology may be influenced by a number of factors including inherited mutations and modifiable exposures, such as socioeconomic deprivation, sleep disruption, and immune‐related variation.^73^ These insights support the need for integrative models that reflect the combined impact of genetic and environmental drivers on disease trajectory.

### 3.1 Environmental and lifestyle modifiers

#### 3.1.1 Role of socioeconomic deprivation

Socioeconomic deprivation is strongly associated with poorer health outcomes, lower life expectancy, and increased dementia risk.^74^, ^75^ In those living with dementia, greater deprivation may worsen long‐term outcomes, shortening survival,^75^, ^76^ and accelerating loss of functional independence.^77^ The link between deprivation and prognosis is primarily explained by the broader social and structural environment, including access to services,^74^ although individual‐level differences in income, education, lifestyle, and health factors may also contribute. Most research into the impact of deprivation on dementia prognosis has focused on AD, or all‐cause dementia, whereas the relationship in FTLD syndromes (comprising behavioral variant FTD [bvFTD], primary progressive aphasia [PPA], PSP, and corticobasal syndrome [CBS]) is unknown. Given differences in disease mechanisms, clinical presentations, and availability of specialist services, deprivation effects in FTLD syndromes may diverge from those observed in AD.

Recent analyses from the PiPPIN study, an epidemiologically based phenotyping study in the United Kingdom,^78^, ^79^, ^80^ investigated whether prognosis is affected by socioeconomic deprivation in the area where people live. Results showed that socioeconomic deprivation was not significantly associated with absolute survival but was associated with earlier institutionalization, with individuals in less‐deprived areas maintaining independent living for longer. This contrasts with findings in AD and all‐cause dementia, where deprivation has been linked to both shorter survival and faster progression. One possible explanation is that access to specialist services within the UK's universal healthcare system may buffer the impact of deprivation on absolute survival, while shortcomings in social care funding and policy disproportionately affect those in deprived areas, contributing to earlier transitions into institutional care.

Thus, these findings underscore the need for stronger social care infrastructure to support people with rare dementia and their caregivers, particularly in deprived settings. Replication in other countries and health system contexts will be critical to determine the generalizability of these findings and to inform policy aimed at reducing inequities in dementia outcomes.

#### 3.1.2 Role of sleep disruption

There is thought to be a bidirectional relationship between sleep and tauopathy,^81^, ^82^ such that: (1) accumulating tau pathology can change features of sleep architecture and timing, and (2) chronic sleep disruptions occurring prior to symptom onset might accelerate the accumulation of tau pathology^83^ and accelerate functional decline. To evaluate the former, PS19 mice carrying an FTD‐associated P301S mutation in the *MAPT* gene^84^, ^85^ were evaluated at 2–3 months of age (early stage) and again at 10 months of age (late stage, shortly before typical death at 10–14 months).

At 2–3 months, when there is only minimal tau hyperphosphorylation in the locus coeruleus and dentate gyrus,^86^ PS19 mice already show decreased sleep spindle density, duration, and power, alongside increased slow oscillation density compared with age‐matched wild‐type controls.^87^ With aging, PS19 mice show marked reduction in both non–rapid eye movement (nREM) and REM duration and continuity, increase in arousal index, further reductions in spindles, and decrease in slow oscillation density. Notably, ∼ 33% of aged PS19 mice develop dream enactment behaviors with increased REM without atonia, resembling REM behavior disorder (RBD). Acute administration of the dual orexin receptor antagonist DORA‐12 resulted in marked increases in non‐REM and REM sleep duration, sleep spindle density and duration, and slow oscillation density, alongside reduction in objective measures of REM without atonia. These findings suggest that electroencephalography (EEG) sleep signatures may emerge in the earliest phases of tauopathy, offering a potential noninvasive biomarker of disease risk. They also warrant future exploration of dual orexin receptor antagonists as a mechanism to improve sleep in tauopathy and possibly mitigate RBD severity.

#### 3.1.3 Endemic parkinsonism: Clusters and risk factors

The nosological term “endemic parkinsonism” establishes those diseases in which the parkinsonian syndrome (typical or atypical) is a dominant feature and, at the same time, they are present only in the unique geographical area.^88^ Currently, there are 10 types of endemic parkinsonism: in the Western Pacific, there are amyotrophic lateral sclerosis/parkinsonism‐dementia complex (ALS/PDC) from the island of Guam, ALS/PDC complex from the Kii peninsula of the Japanese island Honshu, and ALS/PDC from the Western Papua–Irian Jaya.^89^ In the Asian–Oceanic region, a dystonia‐parkinsonism complex from the Philippine island of Panay (X‐linked dystonia–parkinsonism [XDP] or Lubag) and the disease from the island of New Caledonia have been described.^90^, ^91^ The “Caribbean parkinsonism” afflicts the inhabitants of Lesser Antilles in the Western Atlantic, on the islands Guadeloupe and Martinique; the majority of these cases suffer from disease resembling PSP.^92^ In Europe, endemic (and typical) PD has been discovered in the Basque province of Spain; other (and hereditary) PD was described in Upper Austria. The cluster of parkinsonism resembling PSP‐P was described in Hauts‐de‐France near to the Belgian border.^93^, ^94^, ^95^ Most recently, a cluster of parkinsonism in South‐Eastern Moravia in the Czech Republic has been described.^88^

The environmental origin of the disease is strongly presumed in Western Pacific types of parkinsonism, in the parkinsonism from the Caribbean Sea, in the New Caledonia disorder, and in the parkinsonism in Haute‐France. Exposure to plant neurotoxins from Cycas species (mainly *Cycas micronesica*) is assumed in the Western Pacific and New Caledonia forms.^96^ Neurotoxins from the Annonaceae species are presumed to be a cause of Caribbean parkinsonism.^97^ Heavy metals and chemical residues in polluted water and soil are thought to be the cause of the cluster in Hauts‐de‐France.^95^ On the other hand, a genetic origin is presumed in the Basque province (R1441G variant of the LRRK2 gene), in Upper Austria (a specific variant in the VPS35 gene), and in XDP (seven specific alterations in the Xq13.1 locus).^98^ Probable polygenic origin has been suggested for the parkinsonism in South‐Eastern Moravia, because new pathological variants were found in the FBOX7, LRRK2, MT‐ATP8, PAK6, VMAT2, and VPS35 genes.^99^ Overall, the current opinion is that the environmental factors have primarily genotoxic effects; therefore, all types of endemic parkinsonism are probably caused by pathological changes in DNA.^88^

### 3.2 Genetic and immune determinants of tauopathies

More than 100 mutations in the *MAPT* gene, most located in exons within the microtubule‐binding region (MTBR), have been identified in familial and sporadic FTLD‐tau, typically following an autosomal dominant inheritance pattern. These mutations alter microtubule‐binding affinity, increase aggregation propensity, or enhance exon 10 splicing, leading to elevated levels of the more fibrillogenic 4R‐tau species.^100^ Rare exon 1 mutations (R5H/L) disrupt the “paperclip” fold that stabilizes soluble tau through interactions between the amino‐terminus and the MTBR.^101^ In addition, the R5H/L mutation is associated with increased translation from the Met1 codon due to destabilization of an RNA structural loop,^102^ resulting in increased tau levels.

Beyond rare mutations, common genetic variation at the *MAPT* locus also influences disease risk. A large ∼1 Mb inversion on chromosome 17q21, encompassing *MAPT* and nearby genes, defines two extended haplotypes: H1 and H2.^103^ The H1 haplotype strongly predisposes to sporadic 4R‐tauopathies, such as PSP and CBD, and to a lesser extent to AD, particularly in *APOE‐e4–*negative individuals.^104^ Conversely, the H2 haplotype, protective in PSP and CBD, increases risk of the 3R‐tauopathy Pick's disease.^105^ Within H1, the H1c sub‐haplotype, marked by the rs242557 promoter polymorphism, is thought to drive risk through elevated total and 4R‐tau expression, potentially also influenced by distinct epigenetic landscapes of the inverted haplotypes.^103^

Complexity of the Chr17q21 inversion extends to three large copy number variations (CNVs) of the telomeric half of the inversion that define at least 10 structural forms of the H1/H2 dichotomy.^106^ The highly polymorphic 218 kb g (gamma) duplication^107^ spans the LRRC37A/ARL17B duplicon and the partial NSF gene (NSFP1). Importantly, H1c is directly correlated with higher copy numbers of the γ duplication.^106^ NSFP1 is actively translated in a dose‐dependent manner, producing a truncated NSF protein that may interfere with the formation of functional NSF hexamers, essential for SNARE‐mediated vesicle fusion. Because tau also inhibits NSF activity,^108^ elevated 4R‐tau expression from the H1 haplotype, combined with increased truncated NSFP1, could converge to impair NSF function. This suggests possible co‐evolution of genetic variants within the H1/H2 clade, with particular implications for 4R‐tauopathies such as PSP and CBD.

Recent advances in GWAS have further identified multiple genetic loci associated with PSP, particularly within European populations. Previous GWAS research highlighted significant associations at several loci containing various genes including *MAPT, STX6, MOBP, EIF2AK3, RUNX2, SCLO1A2, DUSP10*, and *IRF4*.^109^, ^110^, ^111^ Building upon this foundation, recent computational analyses have further refined these genetic associations. Notably, variations in copy number of the *C4A* gene within the 6p21.32 region have emerged as influential factors contributing to disease risk, with evidence indicating substantial variability across populations.^112^, ^113^, ^114^ Additionally, a meta‐analysis incorporating data from Spanish and Portuguese cohorts alongside previously published European datasets identified a novel genetic signal significantly associated with PSP near *NFASC* and *CNTN2*.^115^ A pilot study in non‐European populations showed that previously identified European‐associated lead single nucleotide polymorphisms (SNPs) exhibit varied odds ratios in other populations, highlighting the critical need for broader, inclusive genetic analyses. Future research initiatives necessitate global collaboration and partnerships, such as with the Global Parkinson's Genetics Program (GP2), to enrich our understanding of PSP's genetic underpinnings across different populations.

Converging evidence points to immune‐related genetic factors as critical modulators of disease susceptibility and progression. In particular, the human leukocyte antigen (HLA) locus—the most genetically variable region in the human genome—is garnering increased attention as a modifier of neurodegenerative disease. Combined analysis of PD and AD identified HLA‐DRB1*04:01 as protective,^116^ whereas studies in PSP implicate distinct risk‐associated HLA variants.^117^ Defining these HLA modifiers may uncover naturally occurring immune mechanisms of risk or resilience that can inform therapeutic strategies.

At the cellular level, cross‐disorder single‐nucleus multiomic analyses of AD, Pick's disease, and PSP have revealed disease‐specific patterns of glial activation and glia–neuron signaling.^118^ These differences primarily involve microglia, astrocytes, and oligodendrocytes, with risk genes most active in divergent microglial states.^118^ In AD, for instance, disorder‐divergent microglia preferentially engage networks of AD risk genes.^118^ These findings suggest that neuroimmune mechanisms are both shared and disease‐specific, with functional genomics providing a bridge between immune cell states and causal genes of therapeutic relevance.

Together, these genetic insights highlight the multifaceted architecture of tauopathy risk, shaped by rare pathogenic variants, common haplotypes, structural genomic complexity, and immune‐related mechanisms. Ongoing integration of genomic, epigenetic, and immune data will be essential to unravel disease mechanisms and identify genetically informed targets for prevention and therapy.

## 4 TOOLS FOR DIAGNOSIS, STAGING, AND PROGNOSIS

Reliable biomarkers for non‐AD tauopathies remain a major unmet need. A critical unresolved question remains as to what is the earliest stage at which tau pathology can be reliably detected, and in which biospecimens this signal first becomes measurable. While AD benefits from a suite of established and emerging biomarkers that help improve diagnostic certainty alongside clinical assessment, conditions such as PSP, CBS, and bvFTD lack validated biomarkers. Recent advances across fluid and imaging modalities, however, are beginning to address this gap.

Extracellular vesicles (EVs) show potential as biomarkers of tau pathology. EVs are released from multiple brain cells to the extracellular space and the blood stream, thereby allowing minimal invasive access.^119^ Importantly, EVs preserve their protein cargo from degradation, a property particularly relevant for tau, which is otherwise rapidly degraded upon release.^120^ Unlike other biofluids, EVs contain a substantial proportion of full‐length tau, facilitating the detection of different tau isoforms.^121^ Moreover, EVs have been implicated in the intercellular transport and propagation of pathological tau aggregates between cells,^122^, ^123^, ^124^ a notion which is further supported by the increased propensity of aggregated proteins to be sorted for EV release.^125^ In large clinical neurodegenerative disease cohorts, plasma EV tau isoforms distinguished PSP and FTD with tau pathology from both healthy controls and other neurodegenerative diseases, highlighting their diagnostic potential.^121^

Complementary insights have been gained from studies of tau's structural domains. The MTBR constitutes the core of tau aggregates across tauopathies, and recent cryogenic electron microscopy (CryoEM) has revealed disease‐specific structural differences in these assemblies.^62^, ^126^, ^127^ Mass spectrometry analysis indicates that, in AD,^128^, ^129^, ^130^ CSF and plasma tau is predominantly truncated within the mid‐domain, generating N‐terminal to mid‐domain fragments,^130^ such as p‐tau217, p‐tau181, and p‐tau205,^128^, ^131^ which strongly correlate with Aβ pathology. By contrast, soluble C‐terminus fragments containing MTBR, including MTBR‐tau243, correlate more directly with tau tangle pathology in AD.^129^, ^132^, ^133^

Extending these findings to primary tauopathies, analyses of brain and CSF revealed enrichment of 4R‐specific MTBR‐tau275 and 282 in PSP, CBD, *MAPT* mutation carriers, and mixed 3R/4R conditions such as AD.^134^ Interestingly, while these fragments are elevated in brain tissue, their CSF levels are reduced in CBD, FTLD‐MAPT, and AD but not PSP. Receiver operating characteristic (ROC) analyses suggested that CSF MTBR‐tau275 and 282 separate CBD from controls and PSP with high accuracy, capturing approximately 80% of autopsy‐confirmed CBD cases regardless of clinical presentations. These findings underscore the potential of MTBR‐based assays to distinguish between tauopathies, refine staging, and capture heterogeneity between sporadic and hereditary forms

Neuroimaging biomarkers provide an additional dimension. Tau PET (tau‐PET) has advanced substantially with the development of next‐generation tracers. Radioligands, such as [^18^F]‐PI‐2620 and [^18^F]‐PM‐PBB3, demonstrate high affinity for 3/4R tau isoforms, but also show significant affinity for 4R tau.^135^, ^136^, ^137^ Albeit some controversy exists regarding their specificity for 4R tauopathies, recent studies have highlighted the potential of [^18^F]‐PI‐2620 to detect 4R tau pathology, showing significantly elevated [^18^F]‐PI‐2620 uptake in target regions (such as basal ganglia and frontal cortex) in clinical cohorts of PSP and CBS.^136^, ^137^, ^138^ This uptake correlates strongly with neuronal AT8‐positive 4R tau aggregates and autoradiography in *post mortem* tissue, respectively.^139^, ^140^ In line with this, elevated radiotracer binding in isolated neurons after injection in tau transgenic PS19 mice revealed that tau‐PET signal predominantly arises from neuronal and oligodendroglial tau aggregates, rather than astroglial sources.^140^ Moreover, the [^18^F]‐PI‐2620 tracer's binding characteristics facilitate discrimination between the 3/4R and 4R tau isoforms through evaluation of region specific distribution volume ratios, perfusion patterns, and tracer tissue clearance.^141^, ^142^ Further, using a data‐driven temporo‐orbital white matter reference enhances ^1^ ^8^F‐PI‐2620 tau‐PET performance in 4R tauopathies, providing superior discrimination compared with traditional cerebellar reference regions.^143^

A first longitudinal [^18^F]‐PI‐2620 imaging study suggests that [^18^F]‐PI‐2620 tau‐PET can track disease progression in 4R tauopathies and can identify potential saturation effects of tau pathology in advanced stages of the diseases.^144^ Further, tau‐PET tracer uptake is associated with clinical decline in CBS patients with 4R tauopathies, underlining its potential as a stratification tool for clinical decision making and clinical trials.^145^ Therefore, neuroimaging with [^18^F]‐PI‐2620 tau‐PET holds promise for a more reliable in vivo diagnosis of 4R tauopathies and shows potential for monitoring and predicting disease progression.

Together, these advances in EV‐derived tau measures, MTBR fragment assays, and tau‐PET imaging provide complementary perspectives on tau pathology in non‐AD tauopathies. Ongoing refinements in assay development and tracer design hold promise for improving diagnostic accuracy, refining staging, and informing prognosis in these heterogeneous and clinically challenging disorders. Across modalities, a central emerging theme is that soluble, dynamic tau species rather than insoluble aggregates alone may represent the earliest and most therapeutically actionable drivers of disease.

## 5 THERAPEUTIC STRATEGIES

The development of tau‐targeted therapies in AD and other tauopathies has gained increasing momentum, although recent trials highlight both opportunities and challenges. Semorinemab, a humanized monoclonal antibody directed against the N‐terminal domain of tau, was evaluated in two Phase 2 studies: Tauriel in prodromal‐to‐mild AD and Lauriet in mild‐to‐moderate AD. While the trials were designed to test whether semorinemab could slow disease progression by limiting the spread of tau pathology, the outcomes differed. In Tauriel, there was no difference in clinical outcomes between placebo and treated patients; However, in Lauriet, semorinemab had a significant effect on cognition despite no measurable effect on tau pathology, functional nor global outcomes, underscoring the complexity of tau‐directed therapeutic approaches.^146^, ^147^

To further interrogate these findings, CSF proteomic analyses were performed using liquid chromatography‐tandem mass spectrometry (LC‐MS/MS). This approach enabled quantification of more than 3,500 proteins, including low‐abundance proteins such as neurofilaments, thereby expanding insight into disease‐associated molecular pathways.^148^ Notably, distinct proteomic signatures were identified between trials, with Lauriet showing enrichment of microglia‐associated proteins (e.g., CXCL10 and CHI3L1), suggesting that immune‐related processes may influence therapeutic outcomes. These data support the hypothesis that microglial pathways, rather than direct tau modification, may modulate clinical responses to tau‐targeted interventions.^149^ Future investigations integrating CSF proteomics with single‐cell approaches may provide mechanistic clarity and inform precision therapeutic strategies, including evaluation of other anti‐tau antibodies, such as bepranemab.

Parallel advances are occurring in non‐AD tauopathies. CBS is a devastating and untreatable neurodegenerative disease that interferes with cognition, movement, with limited therapeutic options and few clinical trials.^150^ CBS is predominantly caused by tau, but two different types of tau can accumulate: 4R‐tauopathy (CBS‐4RT) or the tau in AD (CBS‐AD).^151^ The ability to discriminate between these etiologies using plasma p‐tau217 has recently been demonstrated,^152^, ^153^ a critical advance given that distinct tauopathies may respond differently to therapeutic interventions.^154^ Building on this diagnostic refinement, the Alzheimer's Association Part the Cloud funded a study evaluating a tau‐directed therapeutic CBS. This initiative reflects a growing effort to extend tau‐targeted interventions beyond AD and into primary tauopathies, where therapeutic development has historically lagged.

### 5.1 E2814: Innovative development of next‐generation anti‐tau therapeutics

Etalanetug (E2814) is a novel immunoglobulin G1 (IgG1) monoclonal antibody targeting the MTBR of tau, a core driver of tau seed formation and propagation in AD. It binds with high affinity to R2 and R4 MTBR epitopes,^155^ which are essential for generating “seed‐competent” tau species that enable the spread of tau pathology in the brain. By binding these tau seeds, etalanetug is intended to inhibit tau aggregation and accelerate its clearance, thereby slowing or preventing downstream accumulation of tau tangles and associated cognitive decline.

Unlike first‐generation anti‐tau antibodies, etalanetug has IgG1 backbone. Full effector function will stimulate microglia resulting in clearance of the tau seeds. Building on this mechanistic rationale, a biomarker‐driven development strategy was implemented to accelerate decision‐making, optimize dosing, and focus clinical evaluation in precise patient populations using innovative clinical study designs in rare genetic populations.

In first‐in‐human studies, etalanetug demonstrated predictable IgG pharmacokinetics, dose‐proportional exposure, a 20‐ to 24‐day half‐life, low immunogenicity, no major safety concerns, and confirmed central nervous system (CNS) penetration.^156^ A subsequent Phase 1b study in a dominantly inherited AD (DIAD) population (NCT04971733) showed dose‐dependent target engagement in CSF (measuring the percentage of MTBR‐tau299 and MTBR‐tau354 bound by etalanetug in CSF), with near‐saturation above ∼ 250 ng/mL and pharmacokinetic/pharmacodynamic modeling indicating that 750–3000 mg doses achieve ∼ 50–80% maximal target engagement.

In the Phase 1b study in DIAD, etalanetug produced robust, dose‐dependent reductions in early and late tau pathology biomarkers, including CSF p‐tau217 (∼ 35%–50%) and MTBR‐tau243 (∼ 50%–75%). These biomarker changes suggest potential attenuation of new tangle formation, consistent with stabilization or reduction in tau PET SUVR observed in three subjects with follow‐up imaging at weeks 60 and 108. Etalanetug Phase 1b study results contrast the natural trajectory of mild‐to‐moderate DIAD individuals where p‐tau217 and MTBR‐tau243 in CSF decrease by only ∼ 5%/year, and tau PET dramatically increases over time after symptom onset.^157^, ^158^

The program leverages a biomarker‐driven strategy and add‐on therapy to lecanemab. Based on complementary mechanisms,^159^, ^160^ the two agents are hypothesized to produce synergistic slowing of disease progression across the AD continuum. Ongoing Phase 2/3 trials in DIAD (NCT01760005) and early AD (NCT06602258) evaluate etalanetug alone or with lecanemab, using tau PET, clinical outcomes (Clinical Dementia Rating scale – Sum of Boxes [CDR‐SB]), and CSF biomarkers as primary endpoints. A noninvasive biomarker‐based enrichment strategy is employed in the NCT06602258 study, which uses p‐tau217 to predict tau PET standardized uptake volume ratio (SUVr) that strongly predicts tau positivity and progression.^161^ Participant selection using this non‐invasive biomarker strategy (PrecivityAD p‐tau217 and Aβ42/40 ratio) enables identification of individuals with both amyloid and tau pathology (A+T+) individuals with early AD while reducing burden, cost, and time associated with PET‐based screening.

Overall, the etalanetug program reflects a highly innovative, biomarker‐informed clinical development strategy. By integrating deep biological understanding, precise biomarker measurement, computational prediction models, and combination with anti‐amyloid therapy, the program advances a precision‐medicine approach across the AD continuum. Etalanetug is positioned as an add‐on to amyloid‐lowering therapy, with both sequential and concurrent dosing strategies under evaluation. These efforts lay the groundwork for more effective, personalized interventions targeting the dual pathologies that drive AD progression.

### 5.2 Next‐generation trials for tauopathies

Tau is a promising therapeutic target, but there are many questions about how best to develop tau therapeutics for human diseases. Master protocol designs are more efficient clinical trial designs than classic parallel clinical designs, because they allow for simultaneous testing of multiple tau therapies in the same trial in the same disease (umbrella or platform trials) or evaluation of a single tau therapy simultaneously in multiple diseases.^162^, ^163^ Master protocols also remain open indefinitely, with the potential to add new therapies over time with the costs or time associated with beginning a new clinical trial.

AD is the most common tauopathy, and it has the advantage of having many informative biomarkers of both soluble and soluble forms of tau available for use in clinical trials. A challenge for use of tau therapies in AD is how to combine new tau therapies with currently approved anti‐amyloid drugs. The National Institutes of Health (NIH) funded Alzheimer's Tau Platform (ATP) is a Phase 2 biomarker proof of concept study that is funded to evaluate at least tau therapies alone or in combination with an anti‐amyloid therapy (donanemab) for the ability to slow the accumulation of insoluble tau as measured by MK2640 tau PET over 2 years of tau therapy. Participants will first receive 6 months of donanemab or placebo followed by tau therapy alone, combination donanemab and tau therapy, or donanemab alone. The first tau therapy is an active immunotherapy (AADVac1), and the second tau therapy will be announced in 2026. Additional tau drugs may be tested in the future. Enrollment will begin in early 2026.

PSP is more strongly linked genetically to tau than AD; however, fewer biomarkers are available for use in tau directed therapy trials than for AD. Far fewer clinical trials have been done for PSP than AD or even the similarly prevalent disease ALS. The PSP Trial Platform (PTP) is a Phase 2 clinical proof of concept study that will evaluate at least three tau or neuroprotective therapies for PSP for 1 year of blinded therapy followed by a 1‐year open label extension.

Together, these studies exemplify the evolving landscape of tau‐targeted therapeutics. Integration of advanced biomarker‐based stratification, such as proteomic signatures and plasma p‐tau217, into trial design not only enhances diagnostic precision but also supports a personalized approach to therapeutic development across primary and secondary tauopathies.

## 6 GLOBAL INITIATIVES AND OBSERVATIONAL PLATFORMS

A growing number of multinational initiatives are working to address geographic‐, economic‐, and population‐level disparities in tauopathy research. These platforms integrate genomics, neuroimaging, cognitive, and behavioral data, and social determinants of health (SDH) to uncover disease mechanisms and advance equitable treatment strategies. Below, we highlight some major efforts in this space to exemplify how global collaborations are reshaping the research landscape for tauopathies, particularly in underrepresented populations.

### 6.1 Multi‐Partner Consortium to Expand Dementia Research in Latin America (ReD‐Lat)

Dementia is becoming a leading cause of disability in Latin America (LA), where prevalence is estimated at 8.5% and projected to rise to 19.3%, affecting nearly 198 million people.^164^, ^165^ However, LA remains substantially underrepresented in global dementia research, particularly in studies exploring the combined impact of genetic ancestry and SDH.^166^

To address this gap, the Multi‐Partner Consortium to Expand Dementia Research in Latin America (ReDLat)^167^ was launched to investigate the interplay between genetic, biological, clinical, and social factors in underserved populations.^168^ ReDLat integrates multimodal data—including neuroimaging, genomics, cognitive assessments, and contextual SDH variables—from over 4000 participants across six LA countries and the United States. Supported by the NIH/NIA, Alzheimer's Association, Rainwater Charitable Foundation, Alector, and Global Brain Health Institute (GBHI), the consortium applies machine learning and ancestry‐informed modeling to characterize dementia phenotypes in highly admixed populations.

Conceived in response to persistent structural and scientific barriers to biomarker research in the region, the initiative highlights how global interest in blood‐based biomarkers remains focused primarily on AD and is largely centered in high‐income countries.^169^, ^170^ Research on FTD biomarkers is nearly absent.^171^, ^172^ These gaps reflect broader challenges across LA, including limited funding, scarce infrastructure, and a shortage of specialized human capital.

Through equitable research frameworks, the consortium fosters multimodal studies including biomarker discovery, regional capacity building, and scientific inclusion. In synergy with initiatives such as the Latin America and Caribbean Consortium on Dementia (LAC‐CD)^165^ and the Latin American Brain Health Institute (BrainLat),^173^ ReDLat contributes to a coordinated regional agenda that advances innovation, research standardization, workforce development, and long‐term impact on brain‐health policy and care.

### 6.2 FTLD Prevention Initiative (FPI) global initiative

The past few decades have witnessed a rising frequency of FTLD diagnosed across the world.^174^ However, advances in research and treatment approaches are limited to studies conducted in Europe or North America.^175^ The FTLD Prevention Initiative (FPI) is a network helping to treat FTLD globally.^176^ The overall aim is to promote clinical trials of new therapies to prevent FTLD. The goals are to (a) create uniform standards for conduct of clinical trials in familial FTLD by harmonizing protocols, (b) create an international database of familial FTLD research participants who might be eligible for clinical trials, and (c) promote responsible data sharing across cohorts.

The FPI brought together the Genetic FTLD Initiative (GENFI) based in Europe and Canada^177^ and ARTFL‐LEFFTDS Longitudinal Frontotemporal Lobar Degeneration (ALLFTD) cohort in North America.^178^ The international FPI has expanded to include ReDLat, Dominantly Inherited Non‐Alzheimer Dementias Study (DINAD), New Zealand Genetic FTD study (FTDGeNZ), Southeast Asia FTD Consortium (SEA‐FTD), Nation‐wide Japanese FTD consortium (FTLD‐J), Longitudinal study of Early onset dementia and Family members (LEAF‐FTD), India FTLD Consortium (IFTD), and the Chinese Familial FTLD Consortium (CFFC). More than 5000 participants from the global cohorts are active in this initiative. A dedicated group of participants and families across the familial FTLD consortia support FPI, and key research projects are ongoing to advance FTLD treatment.^179^

The India FTLD consortium is an exemplar of the global reach of FPI. Using harmonized clinical and cognitive tools across sociolinguistically diverse cohorts,^180^ familial FTLD is reported in 9.5 to 40% of patients.^181^ Several genetic mutations, both pathogenic and variants of unknown significance are reported, including *GRN, MAPT, C9orf72, TBK1, CSF1R*, and *TREM2*. The protective role of life‐course factors, such as education, bilingualism, and social engagement are also emerging from the India FTLD consortium. Findings from countries like India provide compelling evidence to increase harmonized global efforts to develop treatments for FTLD.

Together, these initiatives underscore the growing momentum toward globally inclusive, harmonized, and biologically informed approaches to tauopathy research. Efforts like ReDLat and FPI are not only expanding our understanding of disease heterogeneity across diverse populations but are also laying the foundation for more equitable access to diagnostics, clinical trials, and emerging therapies worldwide.

## 7 SUMMARY

Recent years have witnessed remarkable progress across multiple domains of tau research, spanning fundamental biology and mechanistic insights, to advances in genetics and risk factors, diagnostics, therapeutics, and global collaboration. Advances in elucidating tau's structural complexity, its interactions with lysosomal and immune pathways, and its propagation across neural networks are converging to clarify mechanisms that drive disease onset and progression. Emerging experimental platforms—such as iPSC models, direct neuronal reprogramming, and living HBSC—are enabling more physiologically relevant approaches to modeling tauopathies and testing interventions. Moreover, the rapid evolution of biomarker science is beginning to close critical diagnostic gaps for non‐AD tauopathies. Therapeutic strategies are being reshaped through biomarker‐informed trial designs, proteomic readouts, and precision stratification, providing a pathway toward more targeted interventions. Importantly, global initiatives such as ReDLat and FPI are broadening the scope of research to include diverse populations and harmonized infrastructures, addressing long‐standing inequities in representation and access. Together, these insights underscore that advancing the field of tauopathies will require integration across disciplines, modalities, and geographies.

## CONFLICT OF INTEREST STATEMENT

All disclosures were collected using ICMJE forms and are reported for the past 36 months.

Luc Buée received grants or contacts from ANR (French National Research Agency), and Alzheimer's Association, Rainwater Charitable Foundation; received support for attending AAIC, ADPD, and from Tau Global Consortium; and reports a patent filed not licensed (WO2020120644 with priority EP18306684.4. Buée L, Danis C, Dupré C, Landrieu I, Hybrigenics. New anti‐tau single domain antibody. December 13, 2018).

Kristin R. Wildsmith reports Eisai (employer) and Alzheimer's Association funded her travel to attend meetings including Global Tau 2025. As an employee of Eisai, Inc. she reports stock or stock options.

Suvarna Alladi reports Travel and accommodation support to attend Global Tau 2025 meeting; and reports the following leadership or fiduciary roles: ISTAART Alzheimer's Association, USA (unpaid), World Dementia Council Trustee (unpaid), and Executive Member ARDSI Hyderabad (unpaid).

Taylor Bertucci received support from NIA U01AG072464, and reports patent pending related to APOE4 vascular phenotypes.

Delphine Boche received funding from the Alzheimer's Association, and Medical Research Council (MRC); support for attending Tau Global Conference 2025, NeuroFrance 2025, and British Neuroscience Association 2025; served on grant review board of Agence Nationale de Research (France), Fondation Vaincre Alzheimer (France), Flemish Research Foundation (Belgium), and Royal Society (UK), and is Editor‐in‐Chief, *Alzheimer's Research & Therapy*.

Kathryn R. Bowles received support from UK Dementia Research Institute [award number UK DRI‐4211] for the present manuscript; and report the following grants Parkinson's UK (G‐2304), Rainwater Charitable Foundation, Medical Research Scotland (PHD‐50673‐2023), CurePSP (688‐2024‐01‐Pathway), BBSRC (APP52763), ARUK (ARUK‐PhD2024‐027), and Royal Society (RG∖R1∖241229).

Adam Boxer reports the following grant funding: NIH U19AG063911, R01AG078457, R01AG073482, R56AG075744, R01AG038791, RF1AG077557, P01AG019724, R01AG071756, U24AG057437; Rainwater Charitable Foundation, Bluefield Project to Cure FTD, GHR Foundation, Alzheimer's Association, Association for Frontotemporal Degeneration, Gates Ventures, Alzheimer's Drug Discovery Foundation, UCSF Parkinson's Spectrum Disorders Center, and the University of California Cures AD Program, Biogen, Eisai, and Regeneron. A.B. received consulting fees from Alector, Alexion, Arrowhead, Arvinas, Biogen, BMS, Eli Lilly, Janssen, Merck, Neurocrine, Novartis, Oligomerix, Ono, Oscotec, Otsuka, Switch and Transposon; has stock/option at Alector, Arvinas and Neurovanda; and is scientific cofounder of Neurovanda.

Jennifer Brummet reports that CurePSP has consulting relationships with pharma companies, where no personal compensation was received; all payments made to the organization. All work‐related conference travel has been paid for by CurePSP. In addition, she serves as a member of the Board of Directors for Health Research Alliance (unpaid).

Karen Duff received support from UK Dementia Research Institute; and has royalties or licenses for USF Mouse model. K.D. reports financial or non‐financial interests at SAB Ceracuity Inc. (now dissolved).

Claire S. Durrant reports the following support for the present manuscript: Race Against Dementia (grant funding), James Dyson Foundation (donation for research), and Alzheimer's Society (donation for research). C.S.D. reports PhD grant from Medical Research Scotland with partnership with Ionis Pharmaceuticals, funding from UK Dementia Research Institute; and funding for industrial project (unrelated to this work) from ONO Pharmaceuticals via the UK DRI. C.S.D.’s travel was paid for attending Race Against Dementia annual conference and UK DRI connectome conferences. C.S.D. is Co‐Editor in Chief of Brain and Neuroscience Advances (The Journal of the British Neuroscience Association), and received antisense oligonucleotides for research purposes from Ionis Pharmaceuticals (in kind contribution).

Kurt Farrell reports the following support for the present manuscript: 01 AG070326, CurePSP 685‐2023‐06‐Pathway, and Rainwater Charitable Foundation / Tau Consortium. K.F. served on scientific Advisory board of CurePSP.

Bess Frost received support from Rainwater Charitable Foundation, NIA R01 AG057896, NIA R01 AG078964, Belfer Neurodegeneration Consortium, Transposon Therapeutics, and NINDS RF1 NS112391; consulting fees from MD Anderson Belfer Neurodegeneration Consortium, and Transposon Therapeutics; and payment or honoraria from 2025 University of Texas Southwestern Seminar. B.F. reports the following support for attending meetings and/or travel: 2024 Alzheimer's Association Research Roundtable, paid travel; 2024 – Dark Genome Symposium, New York City, NY, paid travel by Rome Therapeutics; Annual – Tau Consortium Investigators Meeting, paid travel; 3x annual: CMND study section, paid effort/travel by NIH; Annual Belfer Neurodegeneration Consortium Science Day, Paid travel; and Tau 2024, Tau 2025, paid travel by Rainwater Foundation. B.F. has patents planned, issued, or pending (US20250082597A1); and is guest Editor of Alzheimer's and Dementia; Member, Funded Investigator, Belfer Neurodegeneration Consortium; and Member, Funded Investigator, Tau Consortium.

Kanta Horie was supported by Eisai fund and NIH/NIA 5R21AG08196102. K.H. may receive income based on technology (METHODS TO DETECT MTBR TAU ISOFORMS AND USE THEREOF) (PCT/US2020/046224) licensed by Washington University to C2N Diagnostics. K.H. reports the following patent: METHODS TO DETECT MTBR TAU ISOFORMS AND USE THEREOF (PCT/US2020/046224). K.H. is an Eisai‐sponsored voluntary research associate professor at Washington University and has received salary from Eisai.

Petr Kaňovský received grant AZV CR No. NW25‐04‐00194; consulting fees from Dompe and Abbvie; payment or honoraria from Ipsen, Abbvie, Merz, and Medtronic; and participated on a Data Safety Monitoring Board or Advisory Board of Abbvie.

Aimee W. Kao received support from NIH U54 P0549981, Rainwater Charitable Foundation/Tau Consoritum, and NIH P30 P0531421; consulting fees from Ono Pharmaceuticals, Eli Lilly & Co., and 4D Molecular Therapeutics; payment or honoraria from Eli Lilly; support for attending meetings from Rainwater Charitable Foundation; and has stock or stock options at Personalis, Inc, Nine Square Therapeutics, and Junevity, Inc.

Maura Malpetti reports the following support for the present manuscript: Race Against Dementia Alzheimer's Research UK Fellowship (2022‐2027), CurePSP Pipeline Grant and NIHR Cambridge BRC. MM received consulting fees from Astex Pharmaceuticals; support for attending meeting from Guarantors of Brain Travel Grant, and ARUK East Network Travel Grant; and served as lead of the Inflammation Special DEMON Group, Member of the PSP Association Research committee, Member of the ISTAART FTD PIA EC, and Member of the ARUK East Network Research Committee.

Huw R. Morris received support from PSP Association and CBD Solutions for the present manuscript. In addition, H.R.M. reports research grants from Michael J Fox Foundation, CBD Solutions, Drake Foundation, Parkinson's UK, Medical Research Council UK, Cure Parkinson's Trust and NIHR. H.R.M. received consulting fees from Roche, Amylyx, Aprinoia, Arvinas, Skyhawk, AI Therapeutics, and Neuron23; lecture fees/honoraria from Kyowa‐Kirin, BMJ, Movement disorders society, Bial, and Calico; and support for attending meetings and/or travel from Michael J Fox Foundation. H.R.M. is a co‐applicant on a patent application related to C9ORF72 ‐ Method for diagnosing a neurodegenerative disease (PCT/GB2012/052140). H.R.M. reports the following leadership or fiduciary role: Cure PSP Association Advisory Board, Association of British Neurologists Movement Disorders Special Interest Group, Association of British Neurologists Neurogenetics Advisory Group.

Carla Palleis received grant funding from the Else‐Kröner‐Fresenius Stiftung and Thiemann Stiftung. C.P. reports the following patent planned, issued or pending: PCT/EP2024/053388: “Oral Phenylbutyrate for Treatment of Human 4‐Repeat Tauopathies”.

Leonard Petrucelli received grants from NIH and Alzheimer's Association for work related to the present manuscript; and reports licensing of pTDP43 mAb and C9orf72‐149R mouse model.

James Rowe received support from NIHR Cambridge Biomedical Research Centre for work related to the present manuscript. In addition, J.R. received grants or contracts from Wellcome Trust, Medical Research Council, Alzheimers Research UK, PSP Association, AstraZeneca, Lilly, GSK, and Janssen; received consulting fees from Akector, Asceneuron, Astronautx, Alzheimer's Research UK, Astex, Aviado Bio, Booster Therapeutics, ClinicalInk, Curasen Therapeutics, Cumulus Neuro, Eisai, Ferrer, SV Health, Prevail, Vesper Bio, and UCB; and served as Chief Scientific Adviser, Alzheimers Research UK; Trustee, Darwin College; Trustee, Guarantors of Brain; and Dementia Goals Program SAB.

Jessica Rexach received support from the Alzheimer's Association, Rainwater Charitable Foundation, NIA, CurePSP, John Douglas French Alzheimer's Foundation, and Ono Pharmaceutical. Also, Tau Global Conference 2025 travel was supported by meeting organizers.

Amy Rommel reports the following: Rainwater Charitable Foundation provided funding and organizational support for the original conference from which this manuscript was developed. The foundation participated in program committee discussions, speaker selection, conference organization, panel moderation, and provided input on manuscript editing and recommendations. The foundation also provides grant funding to some speakers/authors. She also serves as Scientific Advisory board for CureMAPTFTD (unpaid).

Sandra M. Sanabria Bohórquez received support for attending the Tau2025 meeting from Alzheimer's Association, CurePSP, and the Rainwater Charitable Foundation.

Chihiro Sato reports the following support for the present manuscript: R21 AG081961, the Tracy Family Stable Isotope Labeling Quantitation Center, the Rainwater Charitable Foundation, Association for Frontotemporal Degeneration, and Barnes Jewish Hospital Foundation (BJHF) pilot grant no. 3945. C.S. received royalties or licenses for methods to detect MTBR tau isoforms and use thereof) licensed by Washington University to C2N Diagnostics.

Michael Schöll reports the following support for the present manuscript: Knut and Alice Wallenberg Foundation (Wallenberg Centre for Molecular and Translational Medicine; KAW2023.0371), the Swedish Research Council (2023‐06188), the European Union's Horizon Europe research and innovation program under grant agreement no 101132933 (AD‐RIDDLE) and 101112145 (PROMINENT), the National Institute of Health (R01 AG081394‐01), Gates Ventures, the National Research Foundation of Korea (RS‐2023‐00263612), the Swedish state under the agreement between the Swedish government and the County Councils, the ALF‐agreement (ALFGBG‐965326), the Swedish Brain Foundation (FO2024‐0372), the Swedish Alzheimer Foundation (AF‐994900), the Sahlgrenska Academy at the University of Gothenburg, the Västra Götaland Region R&D (VGFOUREG‐995510) and Innovation platforms, Sahlgrenska Science Park and the National Institute for Health and Care Research University College London Hospitals Biomedical Research Centre. M.S received research support from Beckman Coulter, Bioarctic, Novo Nordisk and Roche; consulting fees from Roche, Lilly, Novo Nordisk; and payment or honorarium from Bioarctic, Lilly, Novo Nordisk, Roche, Triolabs. He is co‐founder and shareholder of Centile Bioscience and serves as Associate Editor with Alzheimer's Research & Therapy. M.S. has served/serves as advisor to Arvakor, Gates Ventures, Eli Lilly and Company, Novo Nordisk, Ribocure, and Roche, received honoraria for presentations or educational material from Bioarctic, Novo Nordisk, Eli Lilly and Company, Roche, and Triolabs, and receives research support (to the institution) from Beckman Coulter, Bioarctic, Gates Ventures, Novo Nordisk, and Roche.

Rohan de Silva received Urso grant from CurePSP; and participation fee and travel and living costs for Tauopathy Challenge Workshop, Chicago, 2024. R.S. is co‐inventor, with UCL Business and Eisai on patents for E2814 / etalanetug and precursors.

Anja Schneider received grants or contracts from tALS Foundation, Schick Foundation and MJFF; participated on a Data Safety Monitoring Board or Advisory Board of CELIA, and Biogen (tau ASO); and reports leadership or fiduciary role in the German Society for Biological Psychiatry.

Lawren VandeVrede received grants from NIH, Alzheimer's Association, and Shenandoah Foundation; consulting fees from Roche, Siemens, Biogen, Lilly, and CND Life Sciences; payment or honoraria from Peerview CME and Haymarket; payment for expert testimony from Peter Lacques; support for attending meetings and/or travel from Biogen, Siemens, and Tau Consortium; and equipment, materials, drugs, medical writing, gifts, or other services from LabCorp, Quanterix, and C2N. L.V.V. participation on a Data Safety Monitoring Board or Advisory Board of NIO‐SILK.

Andrew W. Varga received support from R01 AG080609, R01 AG056682, R01 AG066870, R2 AG088736, Alzheimer's Association grant 2018‐AARG‐589632, and Merck Investigator Studies Program (MISP); and consulting fees from Merck Pharmaceuticals, Jazz Pharmaceuticals, Eisai Pharmaceuticals, and Axsome Therapeutics.

Jacob W. Vogel received support from Knut and Alice Wallenberg Foundation (KAW 2020.0239), and European Research Council (StG‐101221737); and consulting fees from Manifest Technologies Inc. J.V. reports stock or stock options at Manifest Technologies Inc.

Felix L. Yeh is a full‐time employee and owns stock in F. Hoffman‐La Roche Ltd.

Andrew S. Yoo received grants from the Cure Alzheimer's fund, NIA/NIH R01AG078964, and EISAI WashU Research Exchange Program.

Simin Mahinrad is a full‐time employee of the Alzheimer's Association. As a full‐time employee of the Alzheimer's Association, all her travel for attending meetings and/or conferences is covered by her employer.

Heather M. Snyder is a full‐time employee of the Alzheimer's Association. She received grants from NIA and CDC. As a full‐time employee of the Alzheimer's Association, all her travel for attending meetings and/or conferences is covered by her employer. She participated in a Data Safety Monitoring Board or Advisory Board of NIA and NINDS‐funded initiatives including DISCOVERY AD and Microbiome AD/ADRD studies. She served as past board member of Health Research Alliance (unpaid); Research Committee of American Heart Association (unpaid); Liaison of Brain Health Council at American Heart Association (unpaid); Women's Brain Health Committee, AARP (unpaid); chair of CDMRP, DoD Alzheimer's and Related Disorders Committee (unpaid) and XPrize Judge (unpaid). Her spouse works for Abbott in an unrelated area.

Maria C. Carrillo is a full‐time employee of the Alzheimer's Association. She has a daughter in the neuroscience program at USC. She reports receiving grants from NIA and CDC. As a full‐time employee of the Alzheimer's Association, all her travel for attending meetings and/or conferences is covered by her employer. She participated in a Data Safety Monitoring Board or Advisory Board of NIA and NINDS‐funded initiatives, including ADSP. She served on the board of GHR Foundation, and Research Committee at the American Heart Association (unpaid).

The remaining authors report no disclosures. Author disclosures are available in the Supporting Information.

## Supporting information



Supporting Information
